# Plant–plant interactions vary greatly along a flooding gradient in a dam-induced riparian habitat

**DOI:** 10.3389/fpls.2023.1290776

**Published:** 2023-11-24

**Authors:** Liu Ying, Wang Yanfeng, Wu Wenzhou, Ding Zhi, Ma Maohua, Huang Ping, Wu Shengjun, Lou Yanjing

**Affiliations:** ^1^ The Three Gorges Institute of Ecological Environment, Chongqing Institute of Green and Intelligent Technology, Chinese Academy of Sciences, Chongqing, China; ^2^ Institute of Tourism and Culture, Chongqing Business Vocational College, Chongqing, China; ^3^ State Key Laboratory of Resources and Environmental Information System, Institute of Geographic Sciences and Natural Resources Research, Chinese Academy of Sciences, Beijing, China; ^4^ Chongqing Engineering Research Center for Remote Sensing Big Data Application, School of Geographical Sciences, Southwest University, Chongqing, China; ^5^ Key Laboratory of Wetland Ecology and Environment, Northeast Institute of Geography and Agroecology, Chinese Academy of Sciences, Changchun, China

**Keywords:** interspecific relationships, stress gradient hypothesis, functional traits, water fluctuation, reservoir riparian zone, Bermuda grass

## Abstract

Plant–plant interactions under extreme environmental stress are still controversial. The stress gradient hypothesis (SGH) proposes that facilitation prevails under extreme environmental stresses, while an alternative view states that facilitation collapses or even switches back to competition at the extreme end of stress gradients. However, how the relationship between plant–plant interaction and periodic extreme flooding stress varies and its underlying mechanism are still unclear in a dam-regulated riparian ecosystem. We established a controlled experiment using two dominant species pairs (*Cynodon dactylon*–*Cyperus rotundus* and *C. dactylon*–*Xanthium sibiricum*) in the water level fluctuating zone of the Three Gorges Dam to examine their growth responses to the periodic extreme flooding stress. The results showed that as flooding stress increased, the competitive effect of *C. dactylon* on *X. sibiricum* shifted to facilitation, whereas the effect of *X. sibiricum* on *C. dactylon* maintained a strong inhibition. The plant height of *X. sibiricum* was the most important driver of the interaction between *X. sibiricum* and *C. dactylon* along the flooding gradient. The net effect of *C. dactylon* on *C. rotundus* shifted from neutral to negative, and the inhibitory effect of *C. rotundus* on *C. dactylon* became stronger at the extreme end of flooding stress. The root biomass of the two species was the key trait regulating their interaction with increasing flooding stress. Overall, the SGH was partially supported along our periodic extreme flooding stress gradient. Aboveground resource (light) might be the dominant factor driving the response of the interaction between annual plants and perennial clonal plants to periodic flooding stress, whereas belowground resource (water and nutrients) was probably the dominant factor for perennial clonal plants. Our study will help to further understand the environmental responses of plant–plant relationships and their regulatory mechanism, and the succession of riparian plant communities under extreme environmental changes, providing a basic theoretical basis and data support for the ecological restoration and management of riparian wetland vegetation.

## 1 Introduction

Plant–plant interactions are widely recognized as key processes in constructing plant communities, shaping species distribution, and maintaining biodiversity (Cardinale et al., 2002; Silknetter et al., 2020) and, thus, are currently among the most active research topics in ecology. Such interactions range from competition to facilitation, and environmental changes exert a crucial role in determining the direction of the interactions as well as their intensity and prevalence in a given habitat (Liancourt et al., 2017; Zhang et al., 2018). Despite this general consensus, the effect of extreme environmental stress on plant–plant interactions is still controversial (Soliveres et al., 2015). A better understanding of plant–plant interactions therefore would be crucial to model accurately the effects of extreme environmental changes on species and community assembly (Tylianakis et al., 2008).

**Figure 1:**
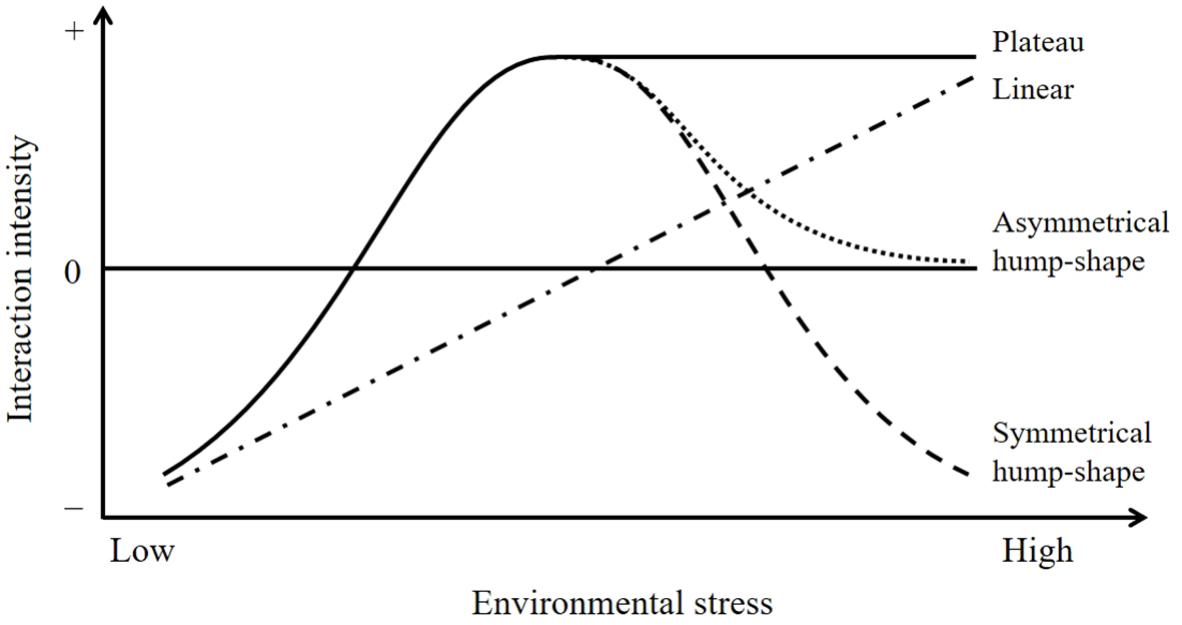
Schematic showing possible variation in the intensity of plant–plant interactions along environmental stress gradient. Facilitative effect may reach an asymptote (plateau), decline to a neutral level (asymmetrical hump shape), or turn into competition (symmetrical hump shape) with increasing environmental stress. Based on figure from Kawai and Tokeshi (2007).

The stress gradient hypothesis (SGH), an enduring theory in ecology, proposes that competition prevails in productive environments, but competition gives way to facilitation with increasing environmental stress (**Figure 1**; Bertness and Callaway, 1994). The original prediction of the SGH (linear model) has been supported by many studies (He et al., 2013; He and Bertness, 2014). However, an alternative view states that facilitation often prevails in intermediately stressful environments and collapses (asymmetrical hump-shape model) or even switches back to competition (symmetrical hump-shape model) at the extreme end of stress gradients, which also has gained empirical support in a variety of natural ecosystems (Maestre et al., 2009; Michalet et al., 2014). The apparently opposing views might stem from inter-study differences in stress types or stress components (single or multiple) or whether the stress gradient is complete. Therefore, more studies need to be conducted to clarify the current debate. According to Burkholder (1952), there are six kinds of interactions between plant species that can grow independently, namely, competition, allelopathy, neutrality, facilitation, reciprocity (or procooperation), and an unnamed interaction (**Table 1**). However, almost all the current studies only consider the unidirectional effects of nursing plants (neighboring plants) on target plants but do not consider the response of nursing plants at the same time, that is, the mutual effects of plants. Hence, how these bidirectional plant–plant interactions respond to the extreme environmental stress remains unclear.

**Table 1:** Simplified presentation of different interactions between two plant species (A and B) that can grow independently, when they meet or do not meet: disadvantage (−), advantage (+), or indifference (0).

	Meeting		Not meeting	
	Species A	Species B	Species A	Species B
Competition	−	−	0	0
Allelopathy	0	−	0	0
Neutrality	0	0	0	0
Facilitation	0	+	0	0
Reciprocity	+	+	0	0
Unnamed	+	−	0	0

Functional traits are proxies for plant physiology, morphology, and phenology and, thus, represent important plant functions such as the ability of light capturing (e.g., plant height), water and nutrient uptake (e.g., root biomass), and reproduction (e.g., ramet number and seed mass; Pérez-Harguindeguy et al., 2013). There is growing consensus that the key functional traits that are the overlaps between response and effect traits can assist in explaining how vegetation-related ecosystem functioning responds to environmental stress (Díaz and Cabido, 2001; Lavorel and Garnier, 2002). Response traits are those that are selected by environmental factors and enable plant persistence under fluctuating abiotic environments (Minden and Kleyer, 2015). For example, the salt marsh plant *Phragmites australis* increases its water use efficiency (leaf δ^13^C) to adapt to saline–alkaline stress and elongates shoot height to resist flooding stress (Liu et al., 2018; Ding et al., 2021). Effect traits are those that influence ecosystem functioning (Minden and Kleyer, 2015; Gustafsson and Norkko, 2019) and are now commonly used to refer to traits that affect plant–plant interactions (Schob et al., 2013; Cameron et al., 2019; Herben et al., 2020). As an example, the plant–plant interactions between three pasture plants, *Alopecurus pratensis*, *Agrostis capillaris*, and *Anthoxanthum odoratum*, can be determined as the product of root traits; i.e., the longer the total root length and the specific root length, the stronger the competition between plants (Minden and Venterink, 2019). Although the key functional traits contain “response-impact” information, few studies have used key functional traits to predict the response of plant–plant interactions to environmental stress.

Riparian habitats, as an ecotone, are fragile due to the frequent disturbances from water fluctuation (Nilsson and Grelsson, 1995; Weissteiner et al., 2016). Moreover, dams on large rivers tend to form extreme periodic terrestrial-to-aquatic switching environments (Wang et al., 2022a). Dam-induced extreme flooding stress in winter leads to the extinction of most original terrestrial vegetation, and only a few plants survived because of their strong flood-tolerant seeds or rhizomes (Guo et al., 2013; You et al., 2017; Guo et al., 2019). When exposed in summer, they grow again, thus forming a new wetland ecosystem that is terrestrial in summer and aquatic in winter. This triggers fundamental changes in the structure and functions of riparian plant communities. Therefore, extreme flooding stress induced by periodic water–land alternation is the most important driver of plant community reconstruction in the terrestrial stage of the water level fluctuating zone (WLFZ). The WLFZ of the Three Gorges Dam (TGD) is the largest riparian zone (349 km^2^) in China, forming on the upper reaches of the Yangtze River after the TGD was completely impounded in 2010 (Lü et al., 2015). Its submerged-exposed fluctuation range is up to 30 m, and it is a typical water–land alternation area (**Figure S1**; Chen et al., 2020). Hence, the WLFZ of the TGD is an ideal place for exploring the effect of periodic extreme flooding stress on plant–plant interactions.

*Cynodon dactylon*, a cushion-forming gramineous plant, is the current pioneer species in the WLFZ of the TGD (Wen et al., 2017). The interactions between *C. dactylon* and other dominant species, such as *Cyperus rotundus* and *Xanthium sibiricum*, affect the structure and succession of plant communities in the WLFZ (Wen et al., 2017). However, little is known about their interspecific interactions along the increasing flooding gradient. Therefore, we examined the growth responses of the two species pairs, *C. dactylon*–*C. rotundus* and *C. dactylon*–*X. sibiricum*, to the flooding stress using three types of plant functional traits related to the ability of light capturing, water and nutrient uptake, and reproduction to answer the following questions: 1) how do the interactions of the two species pairs change, and (2) which types of traits mediate the interactions along the flooding stress gradient? First, based on the niche overlap of the three species (Guo et al., 2018), we hypothesized that extreme flooding stress promoted negative but weak plant–plant interactions, either unidirectional or bidirectional, which differed from the SGH. Second, since *C. dactylon* is a cushion plant and *X. sibiricum* is a tall plant, the two plants might regulate the interspecific interaction by regulating the aboveground traits related to light acquisition. However, *C. rotundus* relies on the clonal propagation of tubers to gain growth advantages (Xu et al., 2021), so its clonal traits may be the key trait.

## 2 Methods

### 2.1 Study area

The study area is located at the upper-mid reaches of Pengxi River, which is one of the largest tributaries of the Yangtze River in China, and covers an area of approximately 55.47 km^2^, accounting for 15.9% of the whole WLFZ of the TGD (Chen et al., 2011). Since the TGD was completely impounded in 2010, the water began to rise in September, reaching the peak level of 150 m above sea level (a.s.l.; 25-m flooding depth above the soil surface) in November, and then starts to recede in the following January, reaching the lowest water level of 175 m a.s.l. (0-m flooding depth) in May (**Figure S1**). Such a hydrological rhythm is contrary to the flooding patterns of natural rivers where flooding occurred in summer. The Pengxi River catchment belongs to a humid subtropical monsoon climate. The mean annual air temperature is 18.6°C, and the mean annual precipitation is approximately 1,300 mm (Wen et al., 2017). The main soil types are Regosols and Anthrosols (FAO Taxonomy; Ran et al., 2021). The vegetation is dominated by *C. dactylon*, *X. sibiricum*, *C. rotundus*, *Bidens frondose*, *Alternanthera philoxeroides*, and *Setaria viridis* (Wang et al., 2022b).

### 2.2 Experimental design

#### 2.2.1 Field survey

A total of 169 quadrats were investigated across six elevation transects, which included *C. dactylon* and *X. sibiricum* or *C. dactylon* and *C. rotundus* or all three species in September 2019, 2020, and 2021 when plants reached their peak cover. Transects were set up every 5 m between elevations of 150 and 175 m. In each transect, three quadrats to five quadrats (1 m × 1 m) were chosen randomly. For the three plant species in each quadrat, the percentage cover (0%–100% vertical projection) was estimated.

#### 2.2.2 Pot experiment

Since the WLFZ of the TGD is flooded in winter and exposed in summer, the stress environment experienced by plants is mainly the flooding stress in winter before plant propagules or seeds germinate. Moreover, the flooding stress and the growth of the plants are separate; that is, plant propagules or seeds experience flooding stress in winter and start to grow as the water recedes. Thus, rhizomes of *C. dactylon*, seedlings of *X. sibiricum*, and tubers of *C. rotundus* were collected across the six elevation transects in May 2020 at the beginning of full exposure of the WLFZ of Baijia River, which is one of the core areas of our study area to achieve the flooding stress condition. Seal rhizomes and tubers were placed in transparent bags with a little tap water to promote sprouting prior to treatments. Seedlings of the three species with a uniform morphology, approximately 2–5 cm high, were planted in pots (20 cm in diameter) with 15-cm-high soil. In order to exclude the influence of different soil physical and chemical characteristics on plant growth, soil within the top 30 cm at 160–165 m altitude was used. The experimental soil was taken from WLFZ of Baijia River and sieved (2 mm) to remove plant roots and debris.

**Figure 2:**
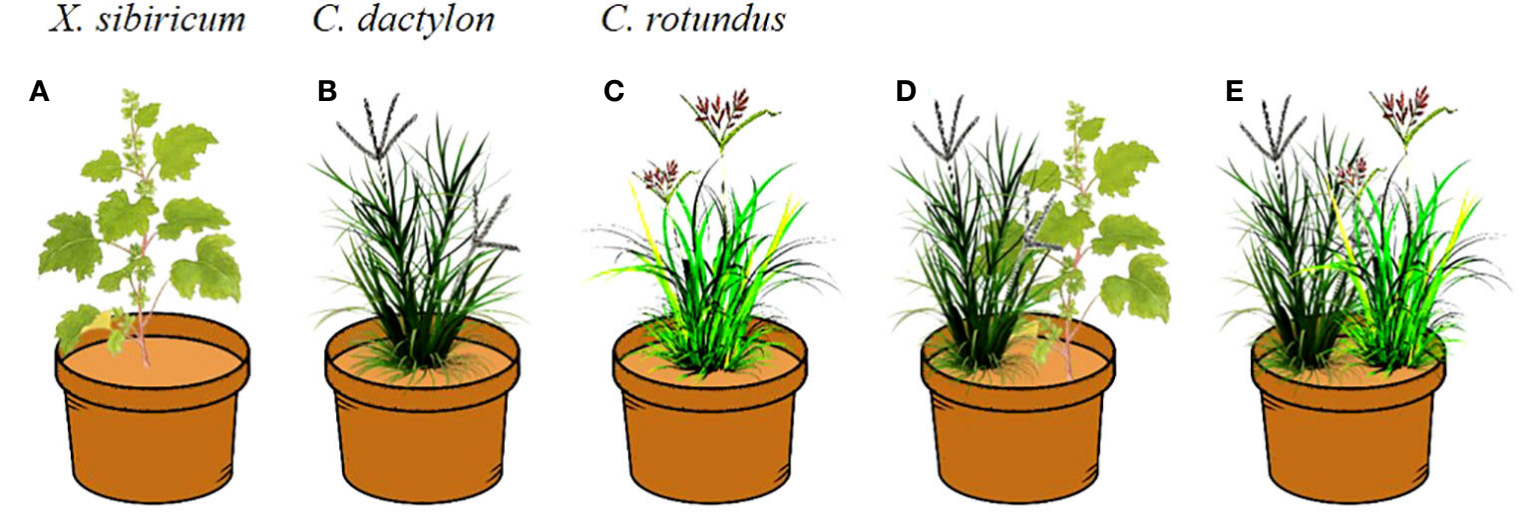
The layout of the pot experiment. For *Xanthium sibiricum*, one seedling per pot **(A, D)**; for *Cynodon dactylon* and *Cyperus rotundus*, five seedlings per pot **(B–E)**.

Our treatments included six elevations (175, 170, 165, 160, 155, and 150 m) and five neighbor effects (monoculture of *X. sibiricum*, *C. dactylon* and *C. rotundus*, mix culture of *X. sibiricum* and *C. dactylon*, and mix culture of *C. rotundus* and *C. dactylon*) with three replications (**Figure 2**). Five seedlings per pot for *C. dactylon* and *C. rotundus* (**Figures 2B−E**) and one for *X. sibiricum* (**Figures 2A, D**) were planted based on our observation in the field. The plants were watered every day to maintain the field capacity. The total number of pots was 90 (5 neighbor effect treatments × 6 elevation treatments × 3 replicates). The pot experiments were conducted in a greenhouse of the Chongqing Institute of Green and Intelligent Technology, Chinese Academy of Sciences.

### 2.3 Measurements

At the end of the experiments (8 weeks later), ramet numbers of *C. dactylon* and *C. rotundus*, lateral length of *C. dactylon*, and height of *X. sibiricum* and *C. rotundus* were recorded. Aboveground and belowground parts of the three plants were sampled and oven-dried at 70°C to constant weight for at least 48 h. The seed mass of *X. sibiricum* and the root biomass of the three plants were measured.

Aboveground and belowground biomass were measured to calculate the relative interaction intensity (RII) according to the following formula:


$$\text{RII}=\left({\text{TB}}_{\text{mix}}-{\text{TB}}_{\text{mon}}\right)/\left({\text{TB}}_{\text{mix}}+{\text{TB}}_{\text{mon}}\right),$$

where TB_mix_ indicates the total biomass of the target plant cultured with the neighbor plant and TB_mon_ indicates the total biomass of the target plant cultured individually. RII was used to assess the interaction between *C. dactylon* and *X. sibiricum*, and *C. rotundus*, with positive values indicating facilitation, negative values indicating inhibition, and zero values indicating no significant interaction (Armas and Pugnaire, 2005). The higher the absolute value of RII, the stronger the interaction between species.

### 2.4 Statistical analyses

The interactive effects of neighbor species and elevation on functional traits and the effects of elevation on RII were tested using ANOVA. Duncan *post-hoc* tests were conducted to examine differences between mono- and mix-cultured species and differences between elevations. Structural equation models were used to explore the relative effects of elevation and plant functional traits on RII using *piecewiseSEM* package in R. Models with an adequate fit (*p* > 0.05) were considered candidate models. Fisher’s *C* statistic and Akaike’s information criterion corrected for small sample size (AICc) were used to evaluate the model (Lefcheck, 2016). The model with the lowest Fisher’s *C* and AICc value was considered the best-fit model. All the above analyses were performed using the software package R 4.1.0 (R Core Team, 2021).

## 3 Results

### 3.1 Variations of cover for *C. dactylon*, *X. sibiricum*, and *C. rotundus* along the elevation gradient

**Figure 3:**
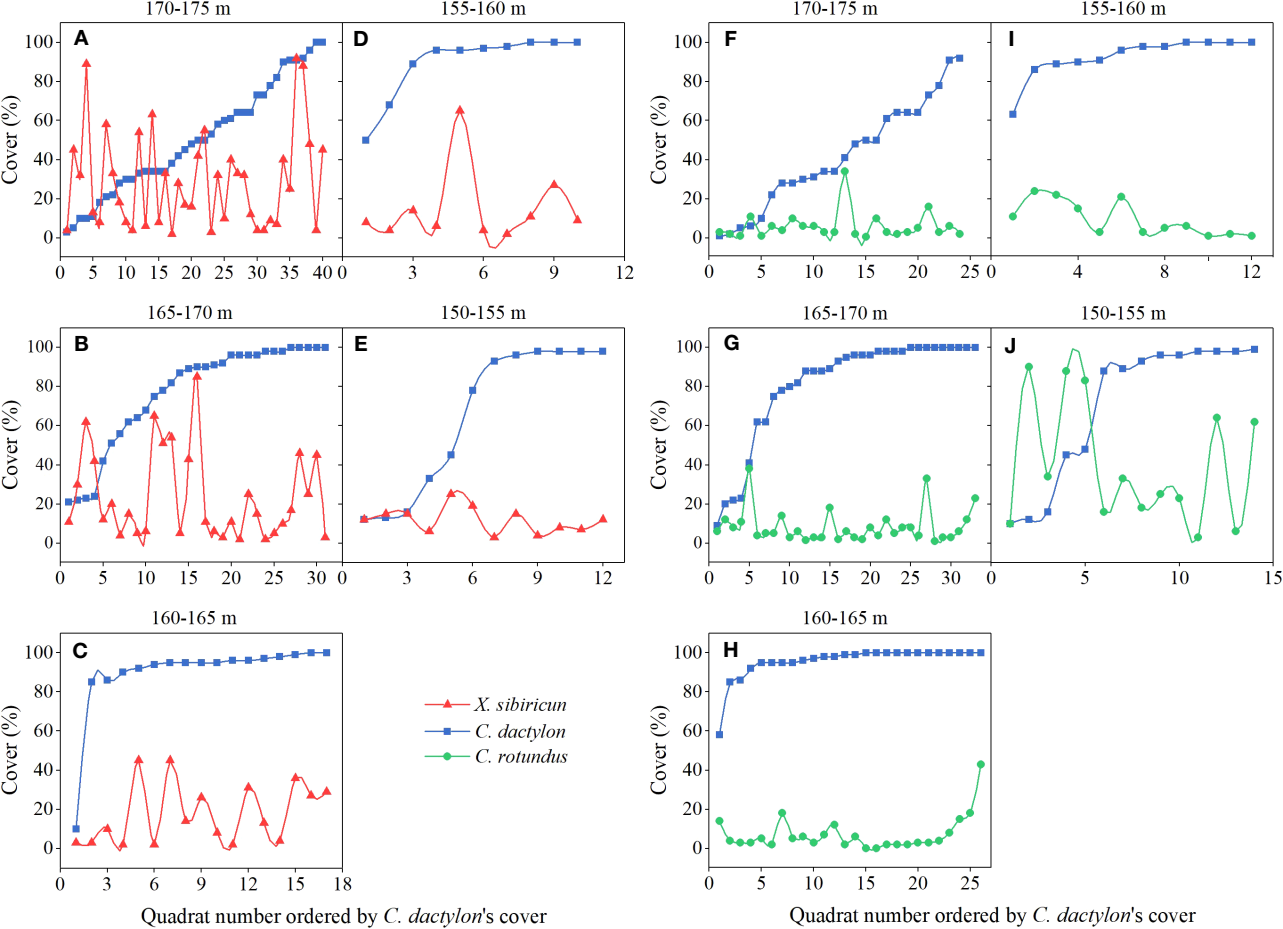
Variation of cover for *Cynodon dactylon* and *Xanthium sibiricum* **(A–E)** and for *C. dactylon* and *Cyperus rotundus* **(F–J)** at different elevation intervals in September 2019−2021.

For species pair *C. dactylon* and *X. sibiricum*, as the cover of *C. dactylon* increased, the change trend of the cover of *X. sibiricum* was not obvious under 165−175-m elevation (**Figures 3A, B**), while its cover decreased or kept at a low level (<20%) under 150−165-m elevation (**Figures 3C−E**), possibly showing an inhibitory effect on *X. sibiricum*. Differing from *X. sibiricum*, the cover of *C. rotundus* decreased or kept a low level (<20%) as the cover of *C. dactylon* increased under 155−175-m elevation (**Figures 3F−I**), possibly showing an inhibitory effect on *C. dactylon*, while its change trend was not obvious under 150−155-m elevation (**Figure 3J**). Due to the uncertainty of the plant–plant interaction and additional factors contributing to their variations in the field, further controlled experiments in the greenhouse were needed to determine the interaction of the two species pairs.

### 3.2 Flooding-induced changes in plant–plant interactions of the two species pairs

**Figure 4:**
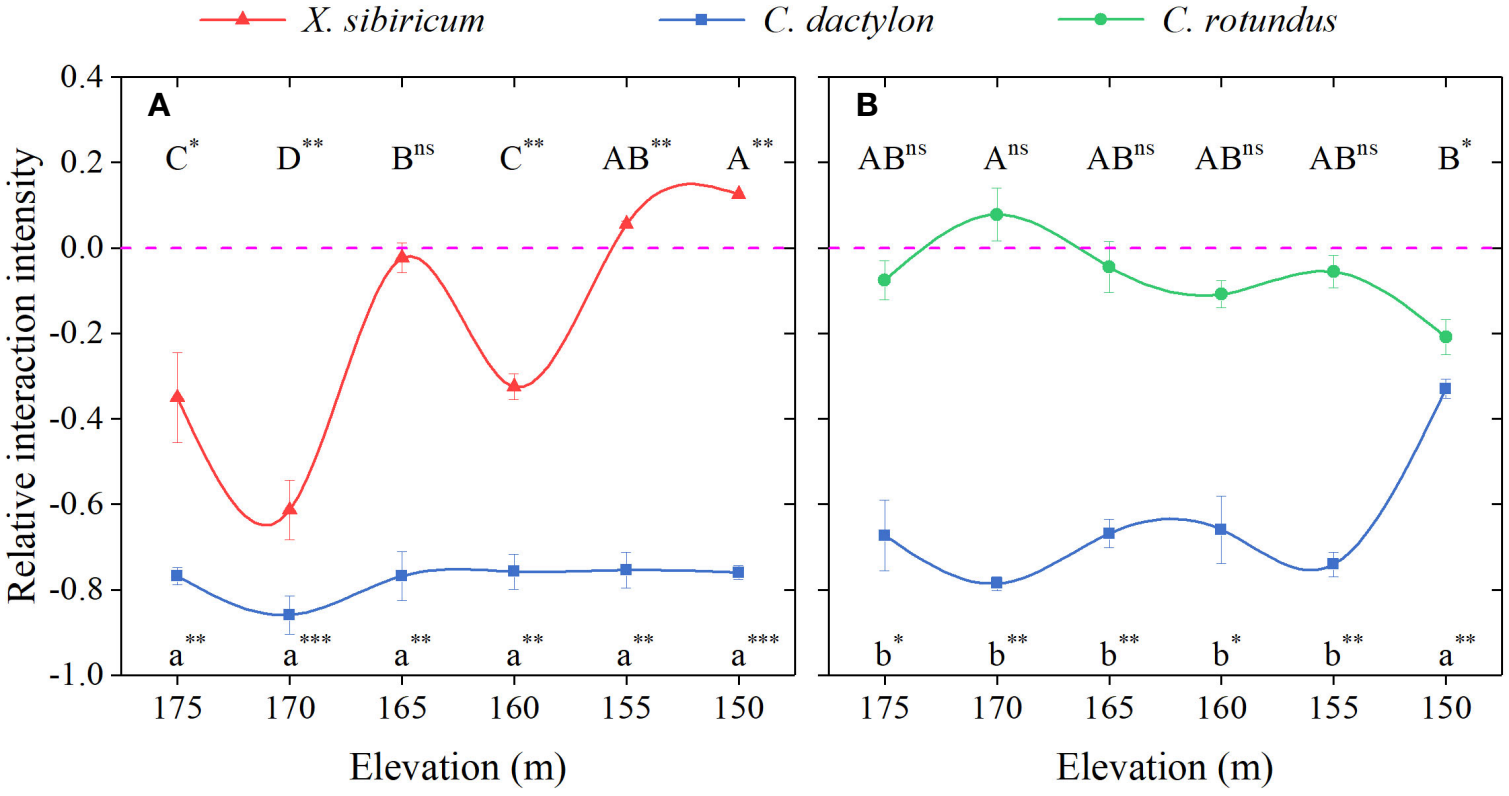
Relative interaction intensity (RII) for total biomass between *Cynodon dactylon* and *Xanthium sibiricum* **(A)** and between *C*. *dactylon* and *Cyperus rotundus* **(B)** at different elevations. Different letters indicate significant differences between elevations (*p* < 0.05). A Duncan *post-hoc* test was used for multiple comparisons. *** represents RII value significantly different from 0 at *p* < 0.001; ** represents significant at *p* < 0.01; * represents significant at *p* < 0.05; ns, not significant.

Extreme flooding stress greatly impacted the interactions between the pioneer species, *C. dactylon*, and the other two dominant species, *X. sibiricum* and *C. rotundus* (**Figure 4**). As flooding stress increased, the inhibitory effect of *C. dactylon* on *X. sibiricum* shifted to a significant boost, whereas the effect of *X. sibiricum* on *C. dactylon* maintained a strong inhibition (RII < −0.75). Thus, the interaction between the two species shifted from competition to the unnamed interaction with increasing flooding stress. The net effect of *C. dactylon* on *C. rotundus* shifted from neutral to negative, and the inhibitory effect of *C. rotundus* on *C. dactylon* became stronger at the extreme end of flooding stress. Thus, the interaction between *C. rotundus* and *C. dactylon* shifted from allelopathy to competition with increasing flooding stress.

### 3.3 Changes induced by neighbor species in plant functional traits of target species along the elevation gradient

**Figure 5:**
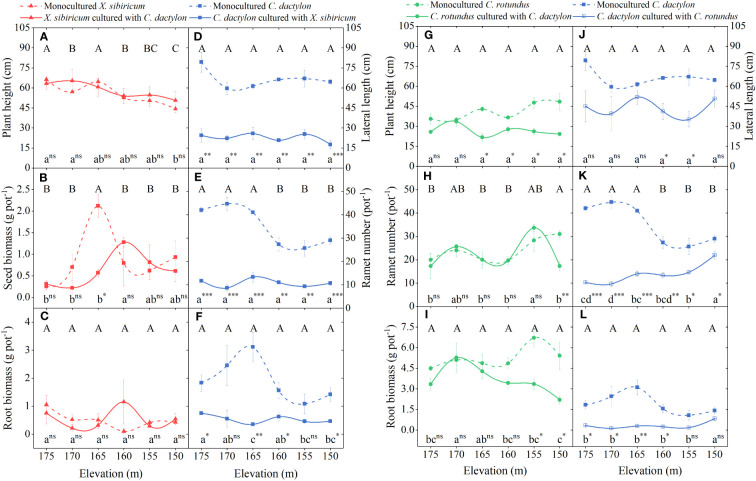
Responses of plant height/lateral length, ramet number/seed biomass, and root biomass of *Xanthium sibiricum* **(A–C)**, *Cynodon dactylon* **(D–F, J–L)**, and *Cyperus rotundus* **(G–I)** to elevation. Values are means ± s.e. (n = 3). Different capital and lower letters denote differences between elevations (*p* < 0.05) for mono- and mix-cultured species. A Duncan *post-hoc* test was used for multiple comparisons. *** represents the value of trait mix-cultured significantly different from that mono-cultured at *p* < 0.001; ** represents significance at *p* < 0.01; * represents significance at *p* < 0.05; ns, not significant.

For *X. sibiricum*, only elevation significantly affected its plant height, which decreased with decreasing flooding stress. The interaction of neighbor species and elevation significantly affected seed mass. Specifically, the seed biomass of mix-cultured *X. sibiricum* was the highest at 160-m elevation but the highest at 165-m elevation for mono-cultured *X. sibiricum*. The effect of neighbor species did not significantly affect any traits (**Figures 5A−C**; **Table 2**). For *C. rotundus*, the neighbor species, *C. dactylon*, significantly decreased its plant height, ramet number, and ramet biomass at low elevations (**Figures 5G−I**; **Table 2**). For *C. dactylon*, neighbor species, either *X. sibiricum* or *C. rotundus*, severely reduced its plant height, ramet number, and ramet biomass and even changed their trends with elevation (**Figures 5D−F, J−L**; **Table 2**).

**Table 2:** Two-way ANOVA results for the effect of neighbor species (N) and elevation (E) on plant height/lateral length, ramet number, and root biomass of *Xanthium sibiricum*, *Cynodon dactylon* cultured with *X. sibiricum* (*C. dac*_*X. sib*), and *Cyperus rotundus* and *C. dactylon* cultured with *C. rotundus* (*C. dac*_*C. rot*).

Traits	Factors	*X. sibiricum*		*C. dac*_*X. sib*		*C. rotundus*		*C. dac*_*C. rot*	
		F	*p*	F	*p*	F	*p*	F	*p*
**Plant height/lateral length (cm)**	N	0.81	0.779	346.28	**<0.001**	65.71	**<0.001**	32.28	**<0.001**
	E	9.92	**<0.001**	2.03	0.110	1.30	0.295	0.93	0.481
	N × E	1.07	0.402	1.54	0.214	4.43	**0.005**	1.03	0.422
**Ramet number (pot^−1^)/seed biomass (g pot^−1^)**	N	3.50	0.073	540.64	**<0.001**	0.93	0.345	414.37	**<0.001**
	E	4.87	**0.003**	12.39	**<0.001**	5.51	**0.002**	7.06	**<0.001**
	N × E	4.25	**0.007**	10.71	**<0.001**	2.70	**0.045**	21.75	**<0.001**
**Root biomass (g pot^−1^)**	N	0.06	0.810	56.17	**0.001**	21.86	**<0.001**	74.74	**0.001**
	E	1.01	0.435	2.73	**0.043**	1.93	0.127	2.63	**0.049**
	N × E	1.53	0.219	3.15	**0.025**	2.89	**0.035**	3.66	**0.013**

F, F test; p, significance of F test.

*P*-Values in bold mean statistically significant effects.

### 3.4 Linkages between plant–plant interactions and plant functional traits

**Figure 6:**
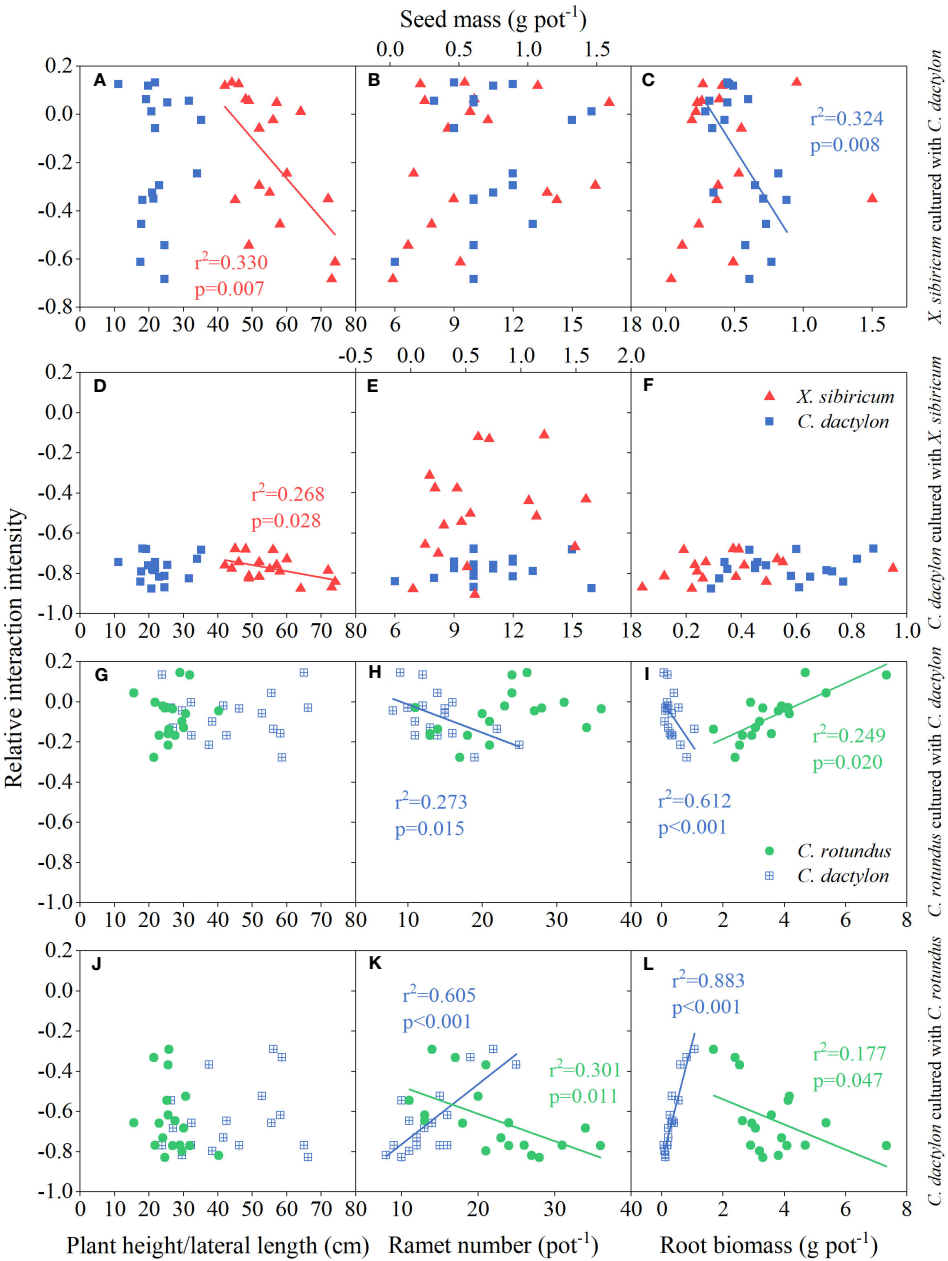
Relationships of relative interaction intensity between *Cynodon dactylon* (blue squares, **A–F**) and *Xanthium sibiricum* (red triangles, **A–F**) and between *C. dactylon* (blue squares with crossing, **G–L**) and *Cyperus rotundus* (green circles, **G–L**) with plant height/lateral length, ramet number/seed biomass, and root biomass.

Our results indicated that RII of *X. sibiricum* cultured with *C. dactylon* exhibited negative correlations with the plant height of *X. sibiricum* and root biomass of *C. dactylon* (all *p* < 0.01; **Figures 6A, C**). Meanwhile, the plant height of *X. sibiricum* was negatively correlated with the RII of *C. dactylon* cultured with *X. sibiricum* (*p* < 0.05; **Figure 6D**). Seed mass of *X. sibiricum* and ramet number and root biomass of *C. dactylon* showed no significant relationships with their RII, respectively (all p > 0.05; **Figures 6B, E, F**). Although ramet number and ramet biomass of *C. dactylon* were negatively correlated with the RII of *C. rotundus* cultured with *C. dactylon*, it exhibited positive correlations with the RII of *C. dactylon* cultured with *C. rotundus* (all *p* < 0.05; **Figures 6H, I, K, L**). On the contrary, ramet number and ramet biomass of *C. rotundus* (except for ramet number) were positively correlated with the RII of *C. rotundus* cultured with *C. dactylon* but negatively correlated with the RII of *C. dactylon* cultured with *C. rotundus* (all *p* < 0.05; **Figures 6H, I, K, L**). Plant height of *C. rotundus* and lateral length of *C. dactylon* were not significantly related to their RII, respectively (all p > 0.05; **Figures 6G, J**).

**Figure 7:**
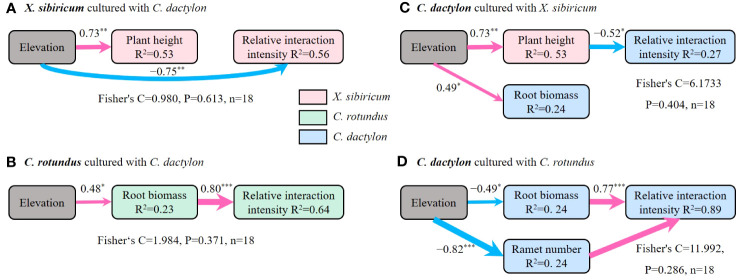
Structural equation models exploring the relative effects of predictors on relative interaction intensity for *Xanthium sibiricum* cultured with *Cynodon dactylon* **(A)**, *Cyperus rotundus* cultured with *C*. *dactylon* **(B)**, and *C*. *dactylon* cultured with *X. sibiricum* **(C)** and *C. rotundus* **(D)**. The width of each arrow is proportional to the strength of the association. The numbers adjacent to the arrows are standardized path coefficients. Red solid lines denote significant positive relationships, and blue denotes significant negative relationships. *** *p* < 0.001, ** *p* < 0.01, and * *p* < 0.05. R^2^ indicates the proportion of the variance explained by predictors in the model. The goodness-of-fit statistics for the structural equation model are shown below each model.

Further analyses showed that, presumably, light was the key factor driving the response of the interspecific interaction between annual plants and perennial clonal plants to flooding stress, whereas water and nutrients were most likely the dominant factors for perennial clonal plants (**Figure 7**). The flooding stress and plant height of *X. sibiricum* co-determined the response of RII of *X. sibiricum* cultured with *C. dactylon* under the flooding stress gradient (**Figures 6A**, **7A**). Similarly, the plant height of *X. sibiricum* was the most important driver of RII of *C. dactylon* cultured with *X. sibiricum* under the flooding stress gradient, explaining 27% of the variation (**Figure 7C**). Root biomass could explain 64% and 89% of the variation in the RII of *C. rotundus* cultured with *C. dactylon* and *C. dactylon* cultured with *C. rotundus* under the flooding stress gradient (**Figures 7B, D**), respectively.

## 4 Discussion

### 4.1 Plant–plant interactions are greatly affected by periodic extreme flooding stress

In our study, the effect of *C. dactylon* on *X. sibiricum* shifted from negative to positive with increasing flooding stress, which supports the original SGH, while *X. sibiricum* maintained a strong inhibition on *C. dactylon*. Thus, the interaction between the two species shifted from competition to the unnamed interaction (one was promoted and the other was inhibited, **Table 1**) with increasing flooding stress. The promotion effect under extreme environmental stresses generally comes from the environmental improvement by neighboring plants, thereby alleviating the harm of environmental stress to the target species. *C. dactylon* is a pioneer species, which facilitates the growth of the plants around by improving soil structure and secreting allelopathic substances (Yuan et al., 2019; Liu et al., 2021). In our pot experiment, the soil was taken from the surface layer of 30 cm at 160–165-m elevation in WLFZ of Baijia River. The site was cultivated land before it was flooded and thus the soil is of good quality. Therefore, we speculate that whether the growth of *X. sibiricum* is inhibited or promoted may be related to the exudate concentration of *C. dactylon*. Although the roots, stems, and leaves of *C. dactylon* can exude allelopathic substances to inhibit the growth of its neighboring plants, low concentrations of exudates have a promoting effect (Yuan et al., 2019). Probably in order to have enough energy to withstand the extreme flooding stress, the high-cost allelopathic effect of *C. dactylon* is weakened, and the concentration of exudates is reduced. Hence, the negative effect of *C. dactylon* on *X. sibiricum* shifted to positive with increasing flooding stress. Our results also indicate that the pioneer species enhanced the resilience of *X. sibiricum* to help them survive together in extreme adversity.

We found that the neutral net effect of *C. dactylon* on *C. rotundus* shifted to negative at the extreme end of flooding stress. Such variation in plant–plant interactions under the flooding stress gradient does not support the original SGH but, instead, is more consistent with the symmetrical hump-shape model. Michalet et al. (2014) stated that the switch to competition was mainly driven by the response of target plants to abiotic stress. However, our result is not driven by the response of *C. rotundus* to flooding stress but by the neighboring plants, *C. dactylon*. This is because whether *C. rotundus* was mono-cultured or mix-cultured with *C. dactylon*, its biomass did not decrease with increasing flooding stress and even increased but was reduced by the neighbor effect at the extreme end of flooding stress (**Figure 5I**). The inhibitory effect of *C. rotundus* on *C. dactylon* changed from strong to weak, which was consistent with the SGH, but *C. rotundus* did not exert the promoting effect on *C. dactylon* under the extreme end of flooding stress, possibly because the stress gradient was incomplete. The strongest flooding stress is 30 m in the WLFZ of the TGD, but the strongest flooding stress is only 25 m in our study area. Further experiments are needed to test whether the interaction between *C. rotundus* and *C. dactylon* will switch to facilitation, neutrality, or even competition under the extreme flooding stress.

Overall, the interaction between *C. rotundus* and *C. dactylon* shifted from allelopathy to competition with increasing flooding stress. However, the inhibition on *C. rotundus* was gradually enhanced, while on *C. dactylon*, it was gradually weakened. This may be because *C. rotundus* can secrete allelopathic substances (Zivanai et al., 2019; Xu et al., 2021), and thereby, the growth of *C. dactylon* has been inhibited. However, the growth of *C. dactylon* was restored under extreme flooding stress (25 m), which may be because the high-cost allelopathic effect of *C. rotundus* is weakened due to energy conservation. Instead, the growth of *C. rotundus* was inhibited by *C. dactylon*. However, although *C. dactylon* can also secrete allelopathic substances (Yuan et al., 2019), it is possible that *C. rotundus* is not affected by its allelopathic substances. Therefore, the effect of *C. dactylon* on *C. rotundus* was neutral under weak flooding stress.

### 4.2 The key traits were different for plants of different life forms

In support of our second hypothesis, plant height of *X. sibiricum* helped predict the interaction between *X. sibiricum* and *C. dactylon* along the flooding stress gradient. Specifically, strong flooding stress resulted in lower plant height of *X. sibiricum*, and the inhibitory effect on both species was subsequently weakened. The decrease in plant height of *X. sibiricum* may be the result of a trade-off between vegetative growth and reproductive growth. *X. sibiricum* is an annual plant, which mainly relies on seed propagation, so during the emergence stage of the WLFZ, it must complete its life history to ensure the continuation of the offspring. However, due to the short exposure time (3–4 months) in high flooding stress sites, *X. sibiricum* may preferentially allocate more resources to reproductive growth at the expense of vegetative growth such as plant height. *X. sibiricum* exhibits this trade-off strategy, which may be the memory function caused by its seeds experiencing high-intensity flooding in winter. However, the reduction of plant height probably also weakened the shading effect of *X. sibiricum*, leading to the weakening of the inhibition between the two species. Interestingly, when *X. sibiricum* was the target species, flooding stress directly affected the RII, and the effect was greater than that of functional traits, whereas, for the other two clonal plants, there was no such direct effect. Perennial clones reproduce mainly on clonal propagules, and it is not necessary for them to complete a life history during the emergence of the WLFZ. Therefore, compared with perennial clonal plants, annual plants are more strongly affected by flooding stress, resulting in their RII being also regulated by the environment. This suggests that for annuals, it is not enough to focus on not only ontogenesis but also the impact of the environment on plant interaction.

The root system not only is an essential organ for absorbing nutrients and water but also helps plants expand underground space through its mechanical action (Konoplenko et al., 2017; Freschet et al., 2021). Our model showed that the key traits were not the ramet number of *C. rotundus* or the lateral length of *C. dactylon* but their ramet biomass, which regulated the interaction between *C. rotundus* and *C. dactylon* along the flooding stress gradient, differing from our second hypothesis. Moreover, their root biomass was related to the flooding stress gradient in the opposite way; i.e., the root biomass of *C. rotundus* was positively correlated with the flooding stress gradient, but that of *C. dactylon* was negatively correlated. This indicates that the interspecific interaction between the two species might not mainly lie in the competition for aboveground living space and resources but for underground space and resources in dam-regulated riparian ecosystems. In addition, although *C. dactylon* had no significant effect on the ramet number of *C. rotundus* (except for 150-m elevation), its ramet number was significantly suppressed by *C. rotundus* and increased with increasing flooding stress. Thus, the ramet number of *C. dactylon* was also the key trait, but that of *C. rotundus* was not. The clonal ramet of *C. rotundus* mainly relies on underground expanded tubers rich in nutrients such as starch and sugar (Xu et al., 2021). These nutrients may be sufficient for *C. rotundus* to produce ramets without competing with *C. dactylon*. However, the rhizomes of *C. dactylon* are low in nutrients and may need to compete with *C. rotundus* for nutrients in the soil to produce ramets. Hence, it is not very surprising that root biomass and ramet number are the most responsive traits and influence the interspecific interaction for the grass family simultaneously and that only root biomass is the key trait for the sedge family in dam-regulated riparian ecosystems.

## 5 Conclusions

In summary, plant–plant interactions varied greatly under extreme flooding stress in WLFZ of the TGD. The interaction between *X. sibiricum* and *C. dactylon* shifted from competition to the unnamed interaction (one was promoted and the other was inhibited), while that between *C. rotundus* and *C. dactylon* shifted from allelopathy to competition with increasing flooding stress. The key traits were different for plants of different life forms. Driving force analysis indicated that the light acquisition trait, plant height, determined the plant–plant interaction that included an annual plant and that the nutrient and water acquisition trait, root biomass, mainly regulated the plant–plant interaction between perennial clonal plants along the flooding stress gradient. Interestingly, when *X. sibiricum* was the target species, flooding stress explained the largest part variation of the plant–plant interaction, suggesting that the annual plant performance in the community was affected by abiotic factors directly more than biotic factors. Our study will help to understand where positive or negative plant–plant interactions prevail and reveal the mechanisms underlying their dynamics under periodic extreme flooding stress in a dam-regulated riparian ecosystem. In addition, a potential issue that might have affected our results is a lack of data on the extreme flooding stress (i.e., 30-m flooding depth) in our study area. In future studies, stronger flooding stress data should be added to further validate our results.

## Data availability statement

The original contributions presented in the study are included in the article/**Supplementary Material**. Further inquiries can be directed to the corresponding author.

## Author contributions

LY: Conceptualization, Data curation, Formal Analysis, Funding acquisition, Investigation, Writing – original draft. YW: Investigation, Project administration, Validation, Writing – review & editing. WW: Resources, Software, Visualization, Writing – review & editing. DZ: Conceptualization, Data curation, Investigation, Writing – original draft. MM: Supervision, Writing – review & editing. HP: Supervision, Writing – review & editing. SW: Funding acquisition, Supervision, Writing – review & editing. YJL: Supervision, Writing – review & editing.
