# Supplementary material for: Plant–plant interactions vary greatly along a flooding gradient in a dam-induced riparian habitat
Source: Front Plant Sci. 2023 Nov 24;14:1290776. doi: 10.3389/fpls.2023.1290776 (PMC10704245; doi:10.3389/fpls.2023.1290776)
Supplement: Supplementary file 1 [file Image_1.pdf]

Figure S1 Water level fluctuation in the Three Gorges Reservoir (A) and schematic diagram for water level fluctuation zone of the Three Gorges Reservoir (B). Data for daily changes in water level were obtained from the China Three Gorges Corporation (http://www.ctg.com.cn). Based on figures from Ran et al. 2021 and Dou et al. 2023.

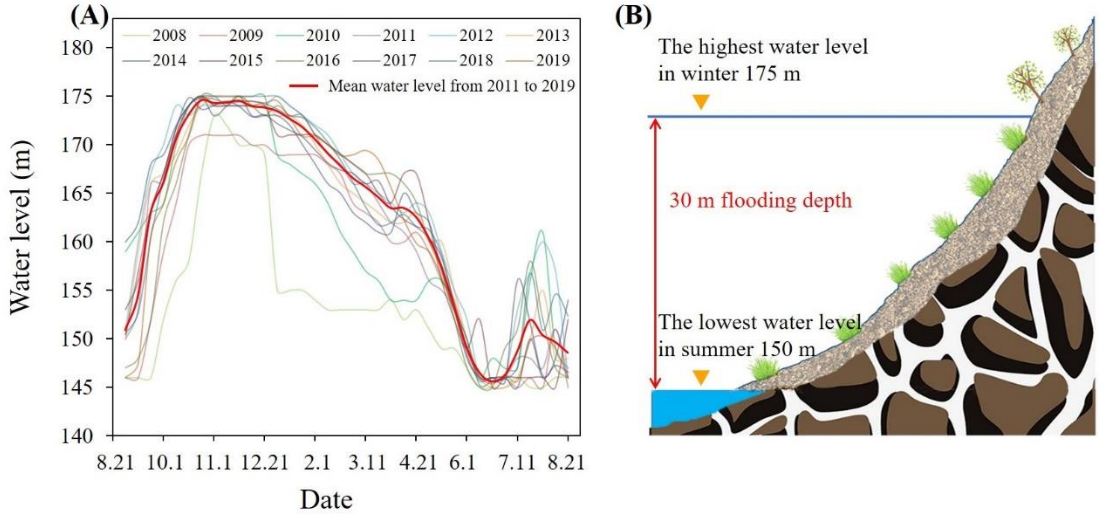

Dou, W., Jia, W., Zhang, J., Yi, X., Wen, Z., Wu, S. et al. (2023) Research progress of vegetation status, adaptiive strategies and ecological restoration in the water-level fluctuation zone of the Three Gorges Reservoir. *Chinese Journal of Ecology*, 2023,42(01),208-218. doi:10.13292/j.1000-4890.202301.018.

Ran, Y., Ma, M., Liu, Y., Zhou, Y., Sun, X., Wu, S. et al. (2021) Hydrological stress regimes regulate effects of binding agents on soil aggregate stability in the riparian zones. *CATENA,* **196,** 104815. doi: 10.1016/j.catena.2020.104815.
